# P-1727. Defining Uncomplicated Candidemia suitable for “shorter is better” Treatment

**DOI:** 10.1093/ofid/ofaf695.1898

**Published:** 2026-01-11

**Authors:** Ilana Reinhold, Susanne Picardi, Blasius Liss, Danila Seidel, Jannik Stemler, Philipp Koehler, Tamara Rügamer, Rosanne Sprute, Oliver A Cornely

**Affiliations:** Faculty of Medicine and University Hospital Cologne, Institute of Translational Research, Cologne Excellence Cluster on Cellular Stress Responses in Aging-Associated Diseases (CECAD), University of Cologne, Cologne, Germany; Department I of Internal Medicine, Faculty of Medicine and University Hospital Cologne, Excellence Center for Medical Mycology (ECMM), University of Cologne, Cologne, Germany, Cologne, Nordrhein-Westfalen, Germany; Department of Anaesthesiology, University Hospital Heidelberg, Heidelberg, Germany, Heidelberg, Baden-Wurttemberg, Germany; Helios University Wuppertal, Wuppertal, Nordrhein-Westfalen, Germany; University Hospital of Cologne, Cologne, Nordrhein-Westfalen, Germany; University of Cologne, Faculty of Medicine and University Hospital Cologne, Department I for Internal Medicine, Excellence Center for Medical Mycology (ECMM), Cologne, NRW, Germany; University of Cologne, Faculty of Medicine and University Hospital Cologne, Institute of Translational Research, Cologne Excellence Cluster on Cellular Stress Responses in Aging-Associated Diseases (CECAD), Cologne, NRW, Germany, Cologne, Nordrhein-Westfalen, Germany; University Hospital Cologne, Cologne, Germany, Cologne, Nordrhein-Westfalen, Germany; Institute for Medical Microbiology, Immunology, and Hygiene, University Hospital Cologne and Faculty of Medicine, University of Cologne, Cologne, Germany, Cologne, Nordrhein-Westfalen, Germany; University Hospital Cologne, Cologne, Nordrhein-Westfalen, Germany; University of Cologne, Faculty of Medicine and University Hospital Cologne, Cologne, Nordrhein-Westfalen, Germany

## Abstract

**Background:**

The success of candidemia treatment depends on patient baseline characteristics, as well as clinical and microbiological findings. While few patient populations with candidaemia are considered at risk for complications, such as neutropenic patients, further stratification criteria distinguishing uncomplicated from complicated disease remain undefined. This literature review aimed to propose criteria for defining uncomplicated candidemia.

**Figure 1:**
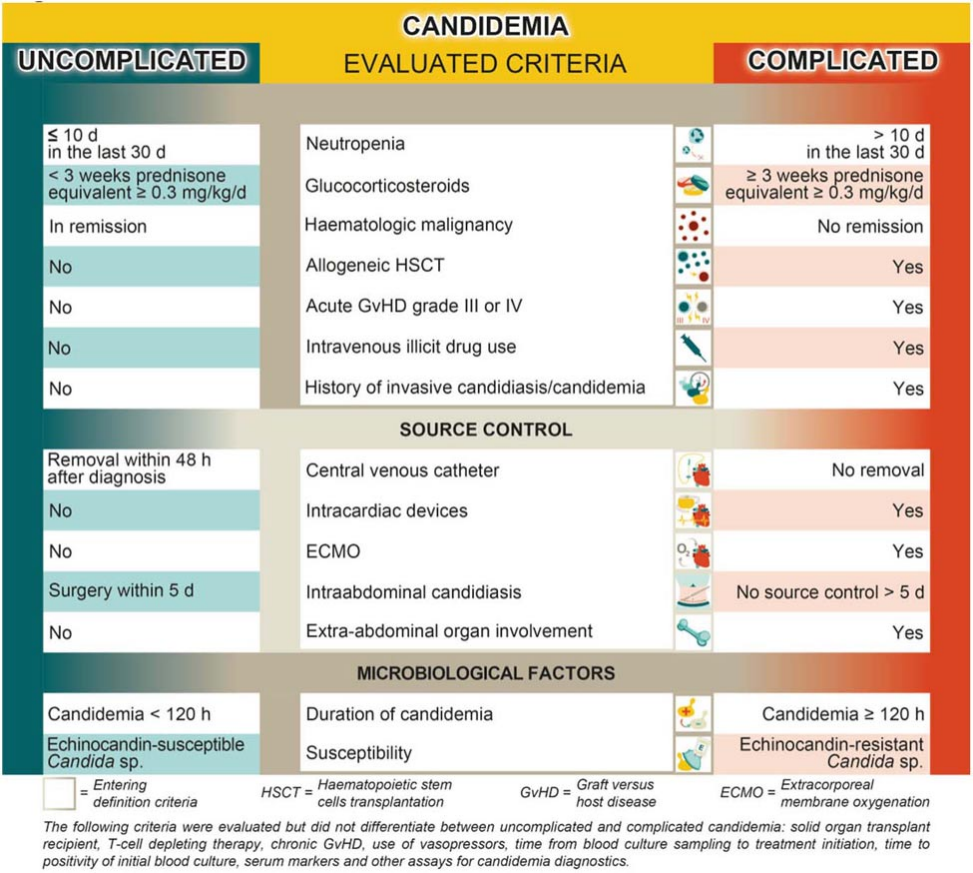
Criteria differentiating uncomplicated and complicated candidaemia.

**Figure 2:**
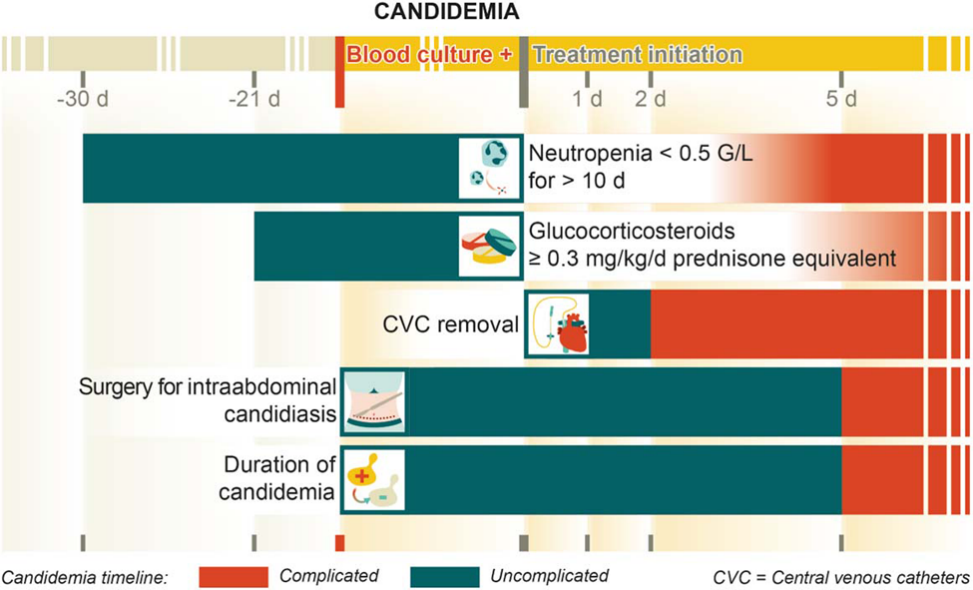
Time-dependent criteria for distinguishing uncomplicated from complicated candidemia.

**Methods:**

A systematic review was performed including literature from 1999 to 2025 to analyse outcome data of different studies and identify criteria associated with favourable or unfavourable outcome. Host factors, source control, time to antifungal treatment initiation, mycological and clinical treatment response, and microbiological findings were assessed.

**Results:**

Uncomplicated candidemia was defined as *Candida* blood stream infection with clearance of blood cultures within five days of adequate antifungal treatment. This includes candidemia with controlled source and requires both mycological and clinical response to treatment within 120 hours. Patients with relevant immunosuppression, i.e. neutropenia, ongoing prolonged use of glucocorticosteroids, haematological malignancy without remission, allogeneic haematopoietic stem cell transplantation or acute graft versus host disease grade III or IV, active intravenous drug use, extra-abdominal organ involvement or candidemia with echinocandin-resistant *Candida* species or recurrent candidemia were considered complicated candidemia (Figure 1). Certain criteria were further distinguished as time-dependent criteria (Figure 2), while other criteria assesed did not enter definition (Figure 1).

**Conclusion:**

This proposed definition for uncomplicated candidemia is based on the first systematic literature review of criteria for a patient population with a favourable outcome of candidemia. To validate and potentially adapt the proposed definition for uncomplicated candidemia for clinical trial use, it must first be applied to large cohorts. Based on this definition, shorter, individualized treatment durations could be considered for selected patient populations.

**Disclosures:**

Blasius Liss, n/a, AstraZeneca: Honoraria|Gilead: Honoraria|GSK: Honoraria|Johnson & Johnson: Honoraria|Moderna: Honoraria Jannik Stemler, MD, AbbVie: Honoraria|Akademie für Infektionsmedizin: Honoraria|Alvea Vax: Advisor/Consultant|Basilea: Grant/Research Support|German Federal Ministry of Education and Research (BMBF): Grant/Research Support|German Society for Infectious Diseases: Travel grants|Gilead: Advisor/Consultant|Gilead: Honoraria|Hikma: Honoraria|Lilly: Honoraria|Meta-Alexander Foundation: Travel grants|Micron Research: Advisor/Consultant|Mundipharma: Honoraria|Noscendo: Grant/Research Support|Pfizer: Honoraria|Scynexis: Grant/Research Support|The Medical Faculty of the University of Cologne: Grant/Research Support Philipp Koehler, n/a, Ambu GmbH: Advisor/Consultant|Ambu GmbH: Board Member|Astellas Pharma: Honoraria|BioRad Laboratories Inc.: Honoraria|Gilead Sciences: Advisor/Consultant|Gilead Sciences: Board Member|Gilead Sciences: Honoraria|Helios Kliniken GmbH: Honoraria|Jazz Pharmaceuticals Germany GmbH: Honoraria|Mundipharma: Advisor/Consultant|Mundipharma: Board Member|Noxxon N.V.: Advisor/Consultant|Noxxon N.V.: Board Member|Pfizer Pharma: Advisor/Consultant|Pfizer Pharma: Board Member|Pfizer Pharma: Honoraria|Sanofi-Aventis Deutschland GmbH: Honoraria Tamara Rügamer, MD, HIKMA: Honoraria Rosanne Sprute, Dr., Hikma: Honoraria|Mundipharma: Honoraria|Pfizer: Honoraria Oliver A. Cornely, Prof. Dr., Al-Jazeera Pharmaceuticals/Hikma: Honoraria|Basilea: Advisor/Consultant|Cidara: Advisor/Consultant|Cidara: Board Member|Cidara: Grant/Research Support|Elion: Advisor/Consultant|F2G: Grant/Research Support|Gilead: Advisor/Consultant|Gilead: Grant/Research Support|Gilead: Honoraria|GlaxoSmithKline: Advisor/Consultant|GlaxoSmithKline: Honoraria|Grupo Biotoscana/United Medical/Knight: Honoraria|Melinta: Advisor/Consultant|Melinta: Board Member|MSD: Honoraria|Mundipharma: Advisor/Consultant|Mundipharma: Grant/Research Support|Mundipharma: Honoraria|Pfizer: Advisor/Consultant|Pfizer: Grant/Research Support|Pfizer: Honoraria|Pulmocide: Board Member|Scynexis: Advisor/Consultant|Scynexis: Grant/Research Support|Shionogi: Advisor/Consultant|Shionogi: Honoraria

